# Sex-Related Differences in Physiological and Biochemical Responses of *Populus nigra* to Bifunctionalized Silver Nanoparticles and Silver Ions Exposure In Vitro

**DOI:** 10.3390/plants14233560

**Published:** 2025-11-21

**Authors:** Valentina Iori, Davide Gentile, Barbara Casentini, Lorenzo Camoni, Anna Fiorillo, Elena Kuzminsky, Iole Venditti, Maria Adelaide Iannelli

**Affiliations:** 1 Institute of Agricultural Biology and Biotechnology-National Research Council (IBBA-CNR), Strada Provinciale 35d, 9, Montelibretti, 00010 Rome, Italy; davide.gentile@uniroma1.it (D.G.); mariaadelaide.iannelli@cnr.it (M.A.I.); 2 Water Research Institute–National Research Council (IRSA-CNR), Strada Provinciale 35d, 9, Montelibretti, 00010 Rome, Italy; barbara.casentini@irsa.cnr.it; 3 Department of Biology, Tor Vergata University of Rome, Via della Ricerca Scientifica, 00133 Rome, Italy; camoni@uniroma2.it (L.C.); anna.fiorillo@uniroma2.it (A.F.); 4 Department for Innovation in Biological, Agro-Food and Forest Systems (DIBAF), University of Tuscia, 01100 Viterbo, Italy; elkuz@unitus.it; 5 Department of Sciences, University of Roma Tre, Via della Vasca Navale 79, 00146 Rome, Italy; iole.venditti@uniroma3.it

**Keywords:** poplar clones, oxidative stress, phytotoxicity, dioecy, nanosilver, glutathione

## Abstract

The aim of this research was to assess the sex-related responses to AgNPs stabilized with citrate (Cit) and glutathione (GSH), relative to silver ions supplied as AgNO_3_ in black poplar (*Populus nigra* L.), a dioecious, woody model species. The impact of the AgNPs-cit-GSH on male and female clones was evaluated by measuring key parameters of oxidative stress. The results showed that exposure to nanosilver resulted in lower Ag accumulation and reduced MDA levels in both genders compared to AgNO_3_. The female clone exhibited a dose-dependent response, characterized by an increase in dry weight (DW), along with a reduction in nutrient uptake, protein content, and ATPase activity, as well as an upregulation of glutathione-S-transferase (GST) activity compared to the control. The male clone displayed a specific treatment response. Exposure to AgNPs-cit-GSH caused a decrease in DW, water content, and nutrient uptake, accompanied by a rise in protein content as well as GST activity. In AgNO_3_-treated male cells, the increase in Ag content and MDA levels corresponded to a decrease in DW and a rise in protein, Cu, and Ca content. These findings offer valuable insights into sexual dimorphism in dioecious woody plants, a topic that has been largely understudied yet is critical for sustainable resource management strategies.

## 1. Introduction

Nanotechnology is a rapidly growing and expanding field that focuses on engineering matters at the nanoscale (1 to 100 nm) to develop novel materials with unique physical, chemical, and biological properties. The applications of nanomaterials are extensive, spanning different sectors, including electronics, energy, medicine, and environmental protection [1,2]. Among the various types available, silver nanoparticles (AgNPs) are the most widely used nanomaterials in fields such as agriculture and plant biotechnology [3]. They are applied to improve seed germination, enhance plant growth and fruit ripening, and as fungicides, nanopesticides, and fertilizers. Due to their well-known antimicrobial, antifungal, and antiviral properties, AgNPs have also been used in medical devices, healthcare, textiles, cosmetics, water purification, food services, and household products [4,5,6]. The widespread use and production of consumer goods containing AgNPs has indeed raised concerns about their nanosafety, as these nanoparticles can be directly or indirectly released into the environment throughout their life cycle, posing a threat to ecosystems and human health. In the environment, AgNPs may undergo physicochemical transformations, leading to the leaching of silver ions (Ag^+^), which are more toxic than the particulate form [2,7].

Several studies have shown that AgNPs can be directly uptaken by plants and bioaccumulated within tissues and organs. Once inside the cells, these nanoparticles can interact with various cellular components, leading to alterations at the morphological, molecular, and physiological levels [2,8,9]. As plants are primary producers and a main entry point for contaminants into the food chain, understanding the phytotoxicity of AgNPs has become a pressing issue. The phytotoxicity of AgNPs is complex and not fully understood due to the interplay of numerous variables, including plant species, NPs characteristics (size, shape, surface coating, concentration), experimental conditions, and exposure time [6,10]. However, a large number of publications have highlighted that AgNPs exposure can induce oxidative stress, cytotoxicity, membrane and DNA damage, protein dysfunction, affecting plant morphology and physiology [2,4,11]. The mechanisms of AgNPs toxicity cannot be attributed solely to the activity of released silver ions (Ag^+^), but the physico-chemical properties of AgNPs are also critical for interactions with plants [12]. For example, several studies have shown that small-sized AgNPs (≤20 nm) exhibit greater toxicity towards plants compared to larger particles, due to their enhanced ability to pass through cell membranes and accumulate in plant tissues [9,13,14]. Similarly, surface coating might change AgNPs properties and affect their toxicity [12]. In a comparative study, Cvjetko et al. [15] reported that cetyltrimethylammonium bromide (CTAB)-coated AgNPs caused a higher toxic effect on *Allium cepa* roots compared to citrate-AgNPs and polyvinylpyrrolidone (PVP)-AgNPs.

To date, majority of studies on the phytotoxicity of AgNPs have focused primarily on food crops, annual herbs, grasses, and flowering plants [16]. Comparatively, fewer reports have investigated the effect of these nanoparticles on woody plants [17,18,19,20]. *Populus* species are widely regarded as a model tree in woody plant studies, due to their fast growth, small genome size, ease of vegetative propagation, and high biomass production [21]. Moreover, *Populus* species are dioecious with male and female reproductive organs on separate individuals. Beyond reproduction, sexual dimorphism also encompasses differences in morphology, physiology, and adaptability to various environments. Males usually tend to allocate more resources to vegetative growth and display a better tolerance to abiotic stress, such as salinity, cold, drought, nutrient deficiency, and heavy metals, compared to females [22,23,24,25]. In this regard, sex-specific responses of dioecious poplars to the long-term effects of AgNPs have not been studied. In this research, we investigated sexual dimorphism in the response to nanosilver using black poplar (*Populus nigra* L.) as a model species. Black poplar is a dioecious, common European tree species, that naturally grows in riparian ecosystems. It is also utilized as a valuable resource in breeding programs to develop high-yielding hybrids that increase biomass production. In previous studies, callus cultures from two black poplar clones—a male clone (Poli) and a female clone (58-861)—were employed to investigate their different adaptive responses to several abiotic stresses [26,27,28,29]. In vitro assays are considered efficient tools for studying stress responses in plants, especially in woody species characterized by long reproductive cycles [30]. Moreover, due to axenic and controlled conditions, plant cell culture allows for the reduction of environmental variations that can influence the bioavailability of toxic substances in the culture medium [31]. Hence, in the current research, callus cultures of *P*. *nigra* clones Poli and 58-861 were exposed for three weeks to nanosilver stabilized with citrate and glutathione (AgNPs-cit-GSH). These novel nanomaterials are designed for environmental applications, including pollution monitoring and remediation [32]. Our aim was to assess the potential phytotoxicity of AgNPs-cit-GSH and determine if poplar female and male clones exhibit differential sensitivity to nanosilver treatment. Phytotoxicity was investigated by evaluating biomass accumulation, silver and nutrient uptake, lipid peroxidation, antioxidant enzyme and ATPase activities relative to the untreated controls. Moreover, this study included exposure to AgNO_3_ to assess the role of released Ag^+^ compared to the surface coating of AgNPs-cit-GSH. By examining the phytotoxicity of AgNPs and sexual dimorphism in black poplar, the research yields significant preliminary insights into the ecological impact assessment within forest ecosystems. As this topic has been poorly characterized [33], these results are pivotal for further studies utilizing realistic experimental designs and for developing sustainable resource management strategies.

## 2. Results

### 2.1. AgNPs-cit-GSH and AgNO_3_ Effect on Poplar Calli Growth

The effects of AgNPs-cit-GSH and AgNO_3_ on the growth of poplar clones were analyzed by evaluating biomass production. As reported in Table 1, with increasing AgNPs-cit-GSH concentrations, the female clone 58-861 showed a significant enhancement in both fresh weight and dry weight (up to 31% and 29%, respectively) compared to the control. On the contrary, the male clone Poli exhibited a marked reduction in both parameters (up to 74% and 54%, respectively) with respect to the control, suggesting a detrimental effect of these nanoparticles on the male clone’s biomass production. In the presence of 2.5 mg/L AgNO_3_, the female clone displayed a decrease both in fresh weight and in dry weight (up to 27% and 12%, respectively) compared to the control, whereas at 5 mg/L a significant increase (up to 25%) in dry weight occurred with respect to the control. In the male clone, long-term exposure to 2.5 and 5 mg/L AgNO_3_ caused a significant reduction in both parameters compared to the control, resulting more pronounced at the lowest concentration.

As reported in Table 1, the female clone 58-861 showed no difference in water content (WC) between the control and either treatment. Conversely, in the male clone, both the AgNPs-cit-GSH and AgNO_3_ treatments significantly reduced the WC compared to the control, though no significant difference was detected between the two treatments.

### 2.2. Oxidative Damage Determination by Lipid Peroxidation

**Figure 1:**
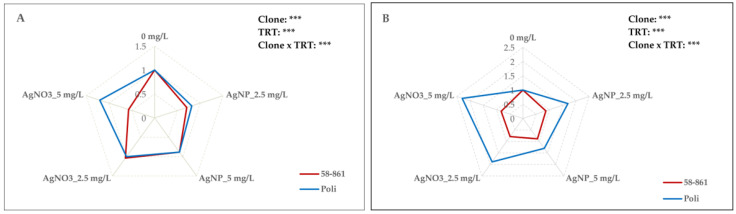
Radar plot representation of lipid peroxidation content (MDA) (**A**) and protein content (**B**) in poplar calli (clones 58-861 and Poli) exposed for three weeks to AgNPs-cit-GSH (2.5 mg/L and 5 mg/L) and AgNO_3_ (2.5 mg/L and 5 mg/L). The data are the average of three biological replicates and report the values with respect to calli grown in the control condition (0 mg/L = control = 1). A detailed Duncan’s test (*p* < 0.05) was performed before normalization and the results are provided in Supplementary Table S1. The analysis of variance for the effect of poplar clone, AgNPs-cit-GSH and AgNO_3_ treatments, and their interaction is reported (*** *p* < 0.001).

MDA measurements revealed significant effects of both sexes and treatments, along with a crucial interaction between them (Figure 1A). AgNPs-cit-GSH treatment, regardless of concentration, did not cause membrane damage to either the male or female clone, as indicated by MDA levels being lower than the respective controls (Table S1). About AgNO_3_, significant differences between sexes were found. A downward trend in MDA was observed in the female clone 58-861 as the AgNO_3_ concentration increased, with a significant reduction observed at 5 mg/L AgNO_3_ (about 43%) compared to the control. Conversely, the male clone Poli showed an increase in MDA, reaching a level 20% higher at 5 mg/L AgNO_3_ relative to the respective control.

### 2.3. Effects of AgNPs-cit-GSH and AgNO_3_ on Protein Content

As reported in Figure 1B, significant effects on protein content due to sex, treatment, and their interaction were found. After three weeks, female callus cultures exhibited a significant decline in total protein content, with a decrease of 13.5% and 11.7% at 2.5 and 5 mg/L AgNPs-cit-GSH, respectively, compared with the control (Table S1). Conversely, callus cultures of Poli displayed a significant increase in protein content, with the most pronounced effect observed at the lowest AgNPs-cit-GSH concentration (up to 69.7%), relative to the control. In the female clone, treatment with AgNO_3_ resulted in a greater reduction in total protein content compared to AgNPs-cit-GSH, with the effect being more pronounced at a concentration of 2.5 mg/L (up to 21.4%). On the contrary, male calli exposed to AgNO_3_ showed a significant, dose-dependent increase in protein content, which was twice that of the control at 5 mg/L.

### 2.4. AgNPs-cit-GSH and AgNO_3_ Effect on Antioxidant Enzyme Activities

**Figure 2:**
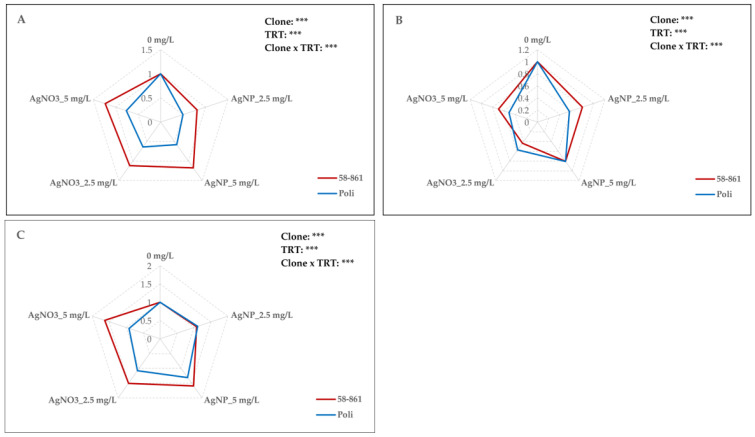
Radar plot representation of catalase (CAT) (**A**), ascorbate peroxidase (APX) (**B**), and glutathione-S-transferase (GST) (**C**) activities in poplar calli (clones 58-861 and Poli) exposed for three weeks to AgNPs-cit-GSH (2.5 mg/L and 5 mg/L) and AgNO_3_ (2.5 mg/L and 5 mg/L). The data are the average of three biological replicates and report the values with respect to calli grown in the control condition (0 mg/L = control = 1). A detailed Duncan’s test (*p* < 0.05) was performed before normalization, and the results are provided in Supplementary Table S2. The analysis of variance for the effect of poplar clone, AgNPs-cit-GSH and AgNO_3_ treatments, and their interaction is reported (*** *p* < 0.001).

As shown in Figure 2, significant effects on antioxidant enzyme activities due to sex, treatment, and their interaction were found. Overall, the female clone consistently displayed higher CAT activity in callus cultures compared to the opposite sex, irrespective of treatment (Figure 2A). The clone 58-861 exhibited a variable response to AgNPs-cit-GSH concentration (Table S2). Specifically, an 18.9% decrease in CAT activity was detected at 2.5 mg/L, followed by a 17.4% increase at 5 mg/L with respect to the control. In the presence of AgNPs-cit-GSH, a decrease in CAT activity compared to the control was observed in the male clone, resulting in 16.4% more activity at 5 mg/L than at 2.5 mg/L, suggesting a concentration-dependent effect even within the overall inhibitory trend.

The clone 58-861 exhibited a slight, dose-dependent enhancement in CAT activity upon AgNO_3_ treatment, with activity at 5 mg/L being 23.4% higher than that of the control. Conversely, exposure to AgNO_3_ caused a reduction in CAT activity in the clone Poli. However, this activity remained higher when compared to the AgNPs-cit-GSH treatment. In particular, at 2.5 mg/L and 5 mg/L AgNO_3_, the results showed increases of 28.4% and 32.2% in CAT activity, respectively, relative to the corresponding AgNPs-cit-GSH concentrations.

The APX enzymatic activity varied between sexes and treatments, as reported in Figure 2B. In the female clone, exposure to AgNPs-cit-GSH resulted in approximately a 20% reduction in APX activity compared to the control, although no statistically significant difference was found between the 2.5 mg/L and 5 mg/L concentrations (Table S2). In the male clone, AgNPs-cit-GSH treatment caused a statistically significant decrease in APX activity relative to the control, resulting in 42% higher at 5 mg/L than at 2.5 mg/L.

In clone 58-861, AgNO_3_ exposure induced a greater reduction in the APX activity compared to the AgNPs-cit-GSH treatment, with the effect being most pronounced at 2.5 mg/L (up to a 56.7% decrease versus the control). The male clone Poli experienced about a 50% decrease in APX activity in the presence of AgNO_3_ compared to the control. Though not statistically significant, a slight difference was observed between the 2.5 mg/L and 5 mg/L AgNO_3_ concentrations.

As reported in Figure 2C, significant effects on GST activity were found due to sex, treatment, and their interaction. In clone 58-861, AgNPs-cit-GSH exposure induced a dose-dependent enhancement of GST activity that reached its maximum value at 5 mg/L compared to the control (Table S2). In the clone Poli, however, an increase in the enzyme’s activity was observed only at the highest AgNPs-cit-GSH concentration (5 mg/L). Exposure to AgNO_3_ induced an enhancement in GST activity in the female clone relative to the control. However, there was no significant difference in enzymatic activity either between the two AgNO_3_ concentrations or when compared to the activity detected at 5 mg/L AgNPs-cit-GSH. Conversely, the male clone Poli showed no significant difference in GST activity compared to the control following any AgNO_3_ treatment.

### 2.5. AgNPs-cit-GSH and AgNO_3_ Effect on Plasma Membrane H^+^-ATPase Activity

**Figure 3:**
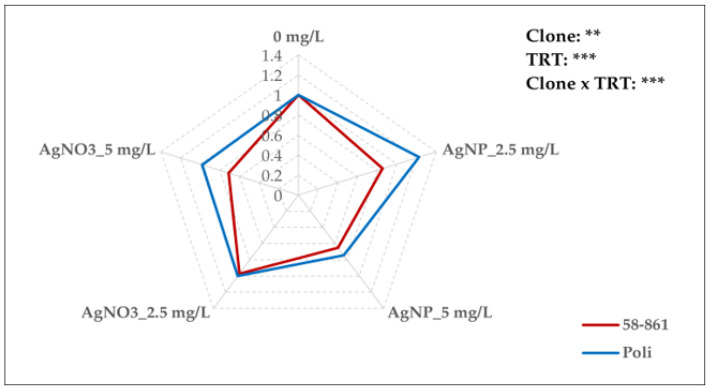
Radar plot representation of ATPase activity in poplar calli (clones 58-861 and Poli) exposed for three weeks to AgNPs-cit-GSH (2.5 mg/L and 5 mg/L) and AgNO_3_ (2.5 mg/L and 5 mg/L). The data are the average of three biological replicates and report the values with respect to calli grown in the control condition (0 mg/L = control = 1). A detailed Duncan’s test (*p* < 0.05) was performed before normalization, and it is provided in Supplementary Table S1. The analysis of variance for the effect of poplar clone, AgNPs-cit-GSH and AgNO_3_ treatments, and their interaction is reported (** *p* < 0.01; *** *p* < 0.001).

As shown in Figure 3, significant effects on H^+^-ATPase activity were found due to sex, treatment, and their interaction. Overall, the male clone consistently exhibited higher H^+^-ATPase activity compared to the opposite gender, regardless of the applied treatment. For the female clone, AgNPs-cit-GSH treatment induced a significant dose-dependent reduction in H^+^-ATPase activity, resulting more pronounced at 5 mg/L (up to 34.8%) compared to the control (Table S1). The male clone, however, displayed a biphasic response to AgNPs-cit-GSH concentrations: ATPase activity increased by 22.6% at 2.5 mg/L, whereas it decreased by 25.5% at 5 mg/L with respect to the control. When exposed to AgNO_3_, the female clone 58-861 showed no statistical differences in H^+^-ATPase activity at 2.5 mg/L, while a 29% decrease was observed at 5 mg/L compared to the control. Notably, AgNO_3_-treated Poli callus cultures displayed no significant differences in plasma membrane H^+^-ATPase across any concentration.

### 2.6. Silver (Ag) Accumulation

As shown in Table 2, Ag content was significantly influenced by sex, treatment, and their interaction. Overall, the male clone showed a greater capacity for Ag accumulation than the female clone. Comparing the two treatments, both the clones Poli and 58-861 accumulated notably higher Ag content when exposed to AgNO_3_. However, the male clone exhibited a higher Ag level at 5 mg/L AgNO_3_ than the female clone at the corresponding concentration. The two clones displayed opposite dose-dependent responses to the nanosilver particles. Specifically, in clone 58-861, Ag content decreased when the AgNPs-cit-GSH concentration was increased from 2.5 to 5 mg/L. Conversely, the clone Poli showed a higher Ag content at 5 mg/L compared to 2.5 mg/L.

### 2.7. AgNPs-cit-GSH and AgNO_3_ Effect on Nutrient Uptake

**Figure 4:**
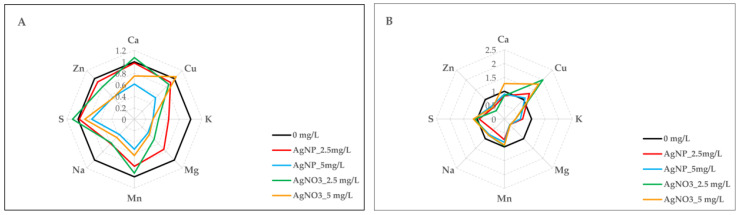
Radar plot representation of nutrient content in poplar calli (clones 58-861 and Poli) exposed for three weeks to AgNPs-cit-GSH (2.5 mg/L and 5 mg/L) (**A**) and AgNO_3_ (2.5 mg/L and 5 mg/L) (**B**). The data are the average of three biological replicates and report the values with respect to calli grown in the control condition (0 mg/L = control = 1). A detailed Duncan’s test (*p* < 0.05) was performed before normalization, and the results are provided in Supplementary Table S3.

Nutrient content was significantly influenced by sex, treatment, and their interaction (Table 3). As reported in Figure 4A and Table S3, the greatest effect on macro- and micronutrient uptake in clone 58-861 was observed following exposure to 5 mg/L AgNPs-cit-GSH, which resulted in a reduction in the concentration of Ca, Cu, K, Mn, Mg, Na, S, and Zn with respect to the control. Similarly, the corresponding AgNO_3_ treatment caused a reduction in nutrient uptake, except for Cu, and S, when compared to the control. In clone Poli, AgNO_3_ affected macro- and micronutrient uptake more significantly than AgNPs-cit-GSH, although differences were observed between the two concentrations (Figure 4B). In detail, at 2.5 mg/L AgNO_3_, a decrease in Ca level was detected compared to the control, although it was not statistically different from the AgNPs-cit-GSH treatment. Conversely, at 5 mg/L, the male poplar cells showed the highest Ca concentration relative to the control. Furthermore, regardless of the concentration, the exposure to AgNO_3_ resulted in the highest levels of Cu and the lowest levels of K compared to the control. Also, the highest S concentration was detected at 5 mg/L AgNO_3_, while the 2.5 mg/L concentration resulted in the lowest Zn level relative to the control. Interestingly, exposure to 2.5 mg/L AgNPs-cit-GSH caused the greatest decrease in both Mn and Na concentrations compared to the control. Finally, a general decrease in Mg levels was detected in the presence of AgNPs-cit-GSH as well as AgNO_3_ relative to the control, although no statistical difference was found between the two treatments.

### 2.8. Principal Component Analysis (PCA)

**Figure 5:**
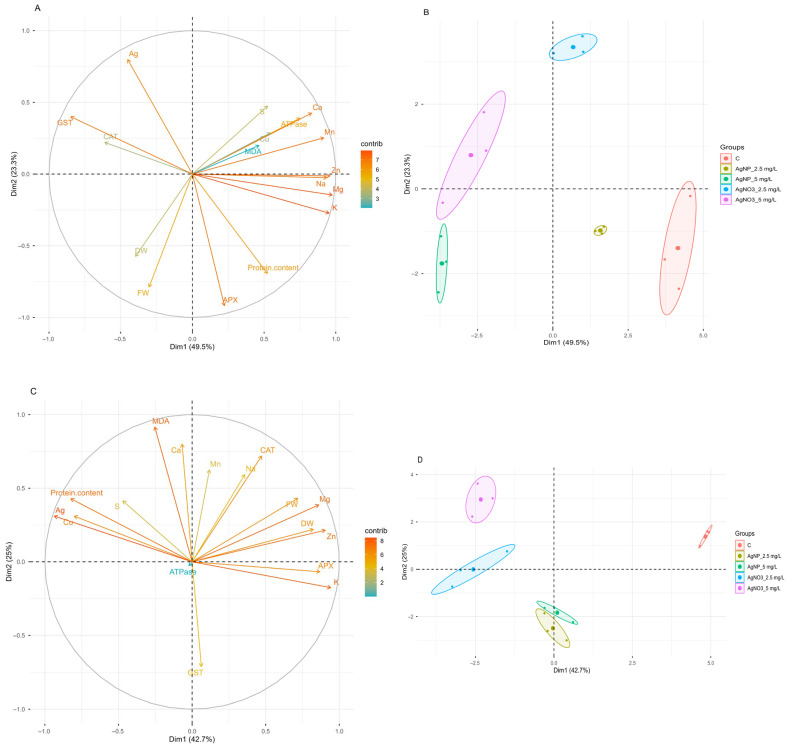
Principal Component Analysis (PCA) of biological responses of clone 58-861 (**A**,**B**) and clone Poli (**C**,**D**) exposed to AgNPs-cit-GSH (2.5 and 5 mg/L) and AgNO_3_ (2.5 and 5 mg/L) for three weeks. (**A**,**C**): Variables plot illustrating the contributions and correlations of measured parameters to the principal components. Vectors indicate the direction and strength of correlation with the dimensions. Colour intensity (blue to orange/red) reflects the variable’s contribution to the displayed components. Variables are: fresh weight (FW), dry weight (DW), protein content, nutrient content (Ca, Cu, Mg, K, Mn, Na, S, Zn), silver (Ag) content, malondyaldehide content (MDA), ascorbate peroxidase (APX), catalase (CAT), glutathione-S-transferase (GST), and ATPase activities; (**B**,**D**) Individuals plot showing the clustering of samples based on treatment. Each point represents an individual replicate, coloured by treatment group (Control (C), AgNPs-cit-GSH 2.5 mg/L, AgNPs-cit-GSH 5 mg/L, AgNO_3_ 2.5 mg/L, AgNO_3_ 5 mg/L). Ellipses represent 95% confidence regions for group centroids.

The concentration of most mineral nutrients and enzymatic activities in clones 58-861 and Poli varied considerably across treatments, with genotype-specific patterns clearly emerging (Figure 5). To explore these differences further, we performed separate principal component analyses (PCA) for each clone. In clone 58-861, the first two components explained 72.8% of the total variance (Dim1: 49.5%; Dim2: 23.3%) (Figure 5A). The first dimension (Dim1) highlighted a concentration-dependent effect ((low vs. high concentration) within each treatment group, whereas the second dimension (Dim2) effectively differentiated between the two treatments. Dim1 displayed a positive correlation with nutrient concentration (mainly Zn, Mg, K, Na, Mn, and Ca) and a negative correlation with GST activity. Conversely, Dim2 provided a distinct separation between AgNPs-cit-GSH and AgNO_3_ treatments. Specifically, the AgNPs-cit-GSH- treated group clustered closer to the control, while AgNO_3_ group was distinctly farther apart, indicating a higher phytotoxicity for the latter (Figure 5B).

In the clone Poli, the first two components explained 67.7% of the total variance (Dim1: 42.7%; Dim2: 25%) (Figure 5C). The variability captured by Dim1 suggested a clear separation of groups based on treatment type rather than a continuous dose–response gradient. Dim1 exhibited a positive correlation with biomass accumulation (FW, DW), antioxidant enzyme activity (APX and CAT), and mineral content (mainly Zn, Mg, and K), while showing a negative correlation with protein, Ag, and Cu content. Dim2 was positively correlated with MDA and Ca content and negatively correlated with GST activity. Samples treated with AgNO_3_ clustered at higher values along this axis, showing higher levels of MDA and protein as well as increased content of Ca and Cu (Figure 5D).

## 3. Discussion

The development of stress tolerance is a complex, adaptive process involving changes across a plant’s genetics, biochemistry, and physiology. Significantly, this process often differs between sexes, reflecting the employment of distinct adaptive strategies. In this study, as highlighted by the PCA, the two clones exhibited differences in their response to the two treatments.

As biomass production is a highly sensitive biomarker for phytotoxicity assessment, the fresh weight and dry weight were measured to determine the effects of nanosilver and AgNO_3_ on cell growth. However, due to the high water content in both poplar clones, only the dry weight was considered. Interestingly, treatment with either AgNPs-cit-GSH or AgNO_3_ resulted in a stimulatory effect on cell growth in the female clone, while in the male clone they induced a reduction in dry weight relative to the control. This inhibitory effect was consistent with the findings reported in our previous study under similar experimental conditions, where the clone Poli was exposed to a different type of AgNPs [20]. These results confirm the male clone’s greater sensitivity to AgNPs compared to the female clone. To date, studies have reported a wide variety of effects of AgNPs on plants, ranging from inhibitory to stimulatory effects at the morphological and physiological levels. This variability mainly depends on the plant species, nanoparticle type, size, concentration, and exposure duration [4,34]. Consistent with findings in clone 58-861, an increase in biomass accumulation was detected in callus cultures of sugarcane (*Saccharum* spp.) treated with 40 and 60 mg/L AgNPs [35] and *Phaseolus vulgaris* L. [36] treated with 50 mg/L AgNPs. Similarly, Dutta Gupta et al. [37], observed that exposure to AgNPs at different concentrations stimulated the growth of rice seedlings. Conversely, several studies reported decreased biomass production upon AgNPs exposure, consistent with findings for the clone Poli. For instance, the addition of AgNPs at different concentrations to a semisolid medium caused a dose-dependent reduction in both fresh weight and dry mass in two apricot cultivars (*Prunus armeniaca* L.) [38]. Furthermore, a significant decrease in both fresh weight and dry mass occurred in pearl millet seedlings (*Pennisetum glaucum* L.) exposed to increasing concentrations of AgNPs [39]. Previous studies converge on the finding that growth reduction in response to adverse conditions is likely an adaptive strategy to maximize plant survival. In particular, when subjected to stress conditions, plants can actively alter their growth patterns by reprogramming both cell division and cell expansion to enhance tolerance [40,41]. For instance, Grodetskaya et al. [42], reported an increase in the activation of the expression of genes responsible for cell wall metabolism, and integrity of cells and cellular structures in birch clones exposed to CuONPs.

The mechanism by which AgNPs reduce biomass production is not yet fully understood, but some studies have demonstrated that these nanoparticles can decrease water uptake in cells by altering the expression of aquaporin genes. For instance, Niemietz and Tyerman [43] demonstrated that Ag^+^ ions can inhibit water transport due to their ability to interact with sulfhydryl groups of aquaporins, affecting the homeostasis of water and other solutes. It is conceivable that in clone Poli, the observed reduction in biomass production is related to the activity of released Ag^+^ ions, as evidenced by the lower water content detected in callus cultures treated with AgNPs-cit-GSH and AgNO_3_ relative to the control. Further studies will be necessary to fully elucidate the complex interaction between aquaporins and the AgNPs-cit-GSH.

It is well known that plant growth and development are related to the uptake of macro- and micronutrients, which is essential for several metabolic pathways [44]. In this research, the female clone showed a significant decline in nutrient uptake in response to both AgNPs-cit-GSH and AgNO_3_ treatments compared to the control, with the effect being most pronounced at the highest concentration (5 mg/L). Specifically, the greatest effect of both treatments was observed in the levels of K, Mn, Mg, Na, and Zn, as evidenced by PCA. Conversely, in the male clone, differences between the two treatments were observed mainly in the uptake of Ca, and S. To date, few studies have focused on the effect of AgNPs on nutrient uptake, and the results are conflicting, as previously reported. For instance, Zuverza-Mena [45] found that at 500 mg/L, AgNPs induced a decline in the content of Ca, Mg, Cu, and Zn in radish sprouts. Similarly, Yang et al. [46] observed a decrease in the levels of Cu and Zn in *Triticum aestivum* exposed to increasing AgNPs concentrations. On the contrary, an enhancement in the content of K, S, and Ca was detected in two cultivars of Oriental lily treated with 100 mg/L AgNPs [47]. It is well known that Ca, K, Mg, and Zn play a crucial role in different plant growth and development processes as well as in protein synthesis and function. Recently, increasing attention is shifting to the role of nutrients in abiotic stress signalling. Several studies have highlighted how changes in the concentrations of Ca, Mg, and K, in particular, are able to trigger various plant responses to stress factors [48,49,50].

Since information on the effect of AgNPs on nutrient uptake is not sufficient and largely contradictory, it remains unclear how these nanoparticles manage to alter nutrient accumulation. In this study, the observed decline in nutrient content may be attributable to the toxic effect of Ag^+^ ions released by AgNPs-cit-GSH and AgNO_3_. It is plausible that nanoAg^0^ and Ag^+^ can alter the synthesis or function of the transporters and protein channels that regulate nutrient flux [51]. In previous research, a down-regulation of genes involved in cation transporters and aquaporins was detected in Arabidopsis exposed to AuNPs. According to the authors, this effect might be a defence mechanism that reduces Au uptake, thereby mitigating phytotoxicity [52], a finding that aligns with our observations regarding the Ag content in the two poplar clones in this study. Both poplar clones showed a significantly lower Ag content upon exposure to both AgNPs-cit-GSH concentrations compared to AgNO_3_, which was consistent with previously published studies [15,53,54]. As reported by Bellingeri et al. [12], the use of the two different capping agents confers high stability to AgNPs-cit-GSH. Consequently, it can be concluded that the Ag remained mainly in the nanoparticle form, and only a minimal amount might have been released from the AgNPs-cit-GSH surface after entering the cells. Interestingly, the two clones showed an opposite trend in Ag accumulation. Further studies on the regulation of genes involved in nutrient transport could help to understand the positive and negative impact of AgNPs on nutrient uptake and plant growth.

The plasma membrane H^+^-ATPase is a key enzyme in several physiological processes, such as nutrient uptake, cell growth and stress response [55]. This membrane protein generates an electrochemical proton gradient by regulating H^+^ influx/efflux across the membrane, thus providing the energy for secondary transport. Changes in the expression of H^+^-ATPase affect the transport of solutes and water across the membrane [56]. In the current study, in the female clone 58-861, the exposure to the highest concentration of both AgNPs-cit-GSH and AgNO_3_ caused a marked reduction in ATPase activity relative to the control. Conversely, in the male clone, ATPase activity showed an increase at 2.5 mg/L AgNPs-cit-GSH, and a marked reduction at 5 mg/L compared to the control. The AgNO_3_ treatment, however, did not affect the enzymatic activity, which remained similar to that of the control. The effect of AgNPs on the synthesis and activity of H^+^-ATPase remains under-investigated. Noori et al. [56] observed that H^+^-ATPase was upregulated upon exposure to both AgNPs and AgNO_3_, although the effect was more pronounced in the presence of the ionic form Ag^+^. It is known that heavy metals induce the expression of H^+^-ATPase, as shown by Janicka-Russak et al. [57] who reported enhanced levels of H^+^- ATPase in cucumber (*Cucumis sativus*) exposed to 10 µM Cd or Cu for six days. The authors suggested that the increased enzymatic activity was due to post-translational modification by reversible phosphorylation of Thr-948 in the protein’s active site, which promoted the formation of a 14-3-3/H^+^-ATPase complex and the creation of a large proton gradient across the membrane. In contrast, our study showed that AgNPs-cit-GSH treatment resulted in a reduction in enzymatic activity, which could be attributed to the inhibition of the phosphorylation mechanism. However, since the regulation of proton pump activity can also occur at the gene expression level, further studies will be necessary to better understand the interaction between AgNPs-cit-GSH and H^+^-ATPase.

Several studies have demonstrated that the toxic effect of AgNPs is related to the production of excess reactive oxygen species (ROS), which leads to oxidative stress in plant cells [2,4,9]. Increased ROS levels cause oxidative stress, leading to metabolic alterations, dysfunctions, and affecting membrane fluidity and permeability. Therefore, maintaining the balance between ROS production and elimination is critical for redox homeostasis and the physiological activities of plants [58]. Lipid peroxidation is recognized as one of the most damaging processes in plants under oxidative stress, resulting from the production of peroxidation byproducts, such as MDA, which leads to the degradation of cell membranes and loss of cellular function [34]. For that reason, MDA is widely used as a biomarker of the extent of lipid peroxidation. As shown by PCA, lipid peroxidation did not emerge as a primary factor differentiating the treatments in the female clone 58-861 under the experimental conditions. Conversely, MDA proved to be a significant stress biomarker in the male clone Poli, effectively distinguishing between the two treatments. Indeed, the MDA level was found to be higher only at the greatest AgNO_3_ concentration compared to the control. Our results suggested that changes in dry weight of both sexes could not be ascribed to lipid peroxidation and that AgNPs-cit-GSH did not cause severe H_2_O_2_ production. While these outcomes are consistent with previous observations in tobacco plants exposed to citrate-coated AgNPs [59], they are in contrast with those reported for the clone Poli treated with AgNPs-Cit-L-Cys under similar experimental conditions, where an enhancement in lipid peroxidation was observed [20]. This variability confirmed that the nanoparticle coating material is a critical factor affecting its phytotoxicity.

Although the AgNPs-cit-GSH induced only mild oxidative stress, ROS, even at low concentrations, triggered downstream stress responses in both poplar clones. The antioxidant defence system of plants comprises several enzymatic components, such as catalase (CAT), glutathione S-transferases (GST), and ascorbate peroxidase (APX). These enzymes play an important role, not only in scavenging free ROS and protecting various cellular components from oxidative stress, but also in a wide range of processes, such as cell growth and division, synthesis of proteins, and cell elongation [60]. As reported by Hasanuzzaman et al. [61], antioxidative defence approaches differ among plant species and genotypes, as well as stress types and duration. In this research, the AgNPs-cit-GSH treatment caused an increase primarily in GST activity in both clones. Conversely, upon exposure to AgNO_3_, GST was up-regulated only in clone 58-861. These results suggested that the GST enzyme plays a more critical role in the response to mild oxidative stress induced by AgNPs-cit-GSH, particularly in the female clone. GST is a crucial enzyme in stress tolerance as it actively binds metal ions via glutathione’s SH group and reduces oxidative stress by preventing the accumulation of H_2_O_2_ and MDA [60,62]. Although GSTs contribute to mitigating damage from several abiotic stressors, including heavy metals, their specific role in the response to AgNPs has been little investigated. For instance, Glavaš Ljubimir et al. [63] observed an enhancement in GST activity in duckweed (*Lemna minor* L.) upon exposure to increasing AgNPs concentrations (0.5–5 mg/L), consistent with our findings. Conversely, the downregulation of CAT and APX enzyme activity has been reported in previous studies. For instance, a decrease in APX and CAT activity was detected in *Allium cepa* roots [15] and *Brassica* seedlings [64] exposed to AgNPs and AgNO_3_.

Furthermore, the male clone might also employ non-enzymatic antioxidants to mitigate the AgNO_3_-induced rise in lipid peroxidation. This is supported by the finding that, unlike the female clone, exposure to both treatments caused an increase in protein content in the male clone, with a more marked effect observed following AgNO_3_ exposure. The PCA showed that this increase was positively correlated with the levels of Cu and S, both of which play important roles in the stress response. S is essential for plant protein synthesis and is a key constituent of several antioxidant compounds that modulate the defence system, while Cu acts as a cofactor for numerous antioxidant and defence-related enzymes [65,66]. Further studies will be necessary to understand the impact of AgNPs-cit-GSH on secondary metabolism.

## 4. Materials and Methods

### 4.1. Synthesis and Characterization of AgNPs-cit-GSH

The AgNPs-cit-GSH, used in this work, were synthesized and functionalized with two capping agents to induce both hydrophilic behaviour and stability in water, respectively, citrate (cit) and glutathione (GSH), following the protocol described in previous work [10,32]. The particle’s nanodimension was characterized using a Shimadzu 2401 PC UV-Vis spectrophotometer (λ_max_ of plasmonic peak at 370–400 nm up to 9 months) and TEM images (Ø = 11 ± 4 nm).

### 4.2. Plant Material and Experimental Setup

Callus cultures of *P. nigra*, the male clone Poli and the female clone 58-861, were obtained by sub-culturing undifferentiated cell clusters from leaf tissue [20]. The experimental treatment was conducted in callus culture conditions. After autoclaving and prior to solidification of the medium, either AgNPs-Cit-GSH or AgNO_3_ stock solutions were added to Murashige and Skoog (MS) medium [67] at concentrations of 0, 2.5, 5 mg/L. To ensure a detectable response in plant cells and assess the potential environmental hazard of the nanoparticles, our experimental design utilized long-term exposure and AgNPs-cit-GSH concentrations, exceeding the predicted environmental range of 0.03 to 0.08 mg/L [2]. For each treatment, five Petri dishes were used, each containing four calli. Petri dishes with callus culture without AgNPs or AgNO_3_ in the medium, served as a control. After three weeks of exposure, for fresh weight (FW) and water content measurements, each callus was collected, washed briefly with sterilized distilled water, dried on filter paper, and weighed. Calli were dried in an oven at 60 °C until constant weight was achieved, yielding the dry weight (DW). Water content was calculated using the formula: 100 × [(FW-DW)/FW]. For biochemical analysis each callus was frozen in liquid N_2_ and stored in a freezer at −80 °C.

### 4.3. Determination of Malondialdehyde (MDA) Content and Antioxidant Enzymatic Activities

The level of lipid peroxidation was determined by measuring malondialdehyde (MDA) content as reported by Iori et al. [20]. Activities of ascorbate peroxidase (APX, EC 1.11.1.11) and catalase (CAT, EC 1.11.1.6) were carried out as described by Iori et al. [20]. Glutathione-S-transferase (GST, EC 2.5.1.18) activity was determined according to Habig and Jakoby [68], using a reaction buffer containing 0.1 M phosphate buffer (pH 6.5), 1 mM reduced glutathione (GSH), 1 mM 1-chloro-2,4-dinitrobenzene (CDNB), and the enzyme extract. The conjugation of CDNB with GSH was monitored at 340 nm (ε = 0.0096 μM^−1^ cm^−1^) at 25 °C using a Thermo Multiskan FC Microplate Photometer (Thermo Fisher Scientific, Waltham, MA, USA). GST activity was expressed as mM CDNB mg protein^−1^ min^−1^. The total soluble protein content was quantified as described by Ernst and Zor [69], using bovine serum albumin (BSA) as a standard.

### 4.4. Purification of Plasma Membranes and H^+^-ATPase Activity

Plasma membranes (PM) were purified from cultured poplar calli by two-phase partitioning, following the procedure described by Fiorillo et al. [70].

The phosphohydrolytic activity of plasma membrane samples was assayed according to Visconti et al. [71]. For each sample, the residual activity in the presence of 0.2 mM of the H^+^-ATPase inhibitor Na_3_VO_4_ was subtracted from the obtained values to calculate the H^+^-ATPase-specific activity [72].

### 4.5. Analysis of Ag and Nutrient Contents

The oven-dried calli were weighed and mineralised. Mineralisation was performed according to Iori et al. [29]. Determination of Ag and nutrient contents was performed using Inductively Coupled Plasma Optical Emission Spectrometry (ICP-OES, 5800 Agilent Technologies, Santa Clara, CA, USA—LOD = 0.02 mg/L).

### 4.6. Statistical Analysis

All results were represented as the mean of three replicates ± standard deviations (SD). All data were checked for normality before analyses of variance by Shapiro–Wilk’s test. Where appropriate, the Box–Cox transformation was performed before statistical analysis. Normally distributed data were processed by two-way ANOVA, using R software (version 4.5.1), followed by Duncan’s test. All statistical tests were considered significant at *p* < 0.05.

Principal Component Analysis (PCA) was performed in R (version 4.5.1) to explore patterns and relationships within the multivariate dataset of biological responses to various treatments in both genders. The dataset comprised quantitative measurements of physiological (FW, DW, Protein content) and biochemical parameters (Nutrient content, Ag content, APX, CAT, MDA, GST, ATPase activity) across five treatment groups (Control, AgNPs-cit-GSH 2.5 mg/L, AgNPs-cit-GSH 5 mg/L, AgNO_3_ 2.5 mg/L, AgNO_3_ 5 mg/L) for both genders.

PCA was then executed using the PCA() function from the “FactoMineR” package. The data was automatically scaled to unit variance (default scale.unit = TRUE) to prevent variables with larger magnitudes from disproportionately influencing the results. Visualization of the PCA outputs was performed using the “factoextra” package. Individual plots (fviz_pca_ind) displayed samples coloured by treatment group, with 95% confidence ellipses. Variable plots (fviz_pca_var) showed the contribution of each parameter to the principal components.

## 5. Conclusions

This study provided novel insight into the sex-specific response of a dioecious woody plant (*P. nigra* L.) to AgNPs and Ag^+^ treatments, a topic that has been poorly characterized yet is important for informing breeding strategies and developing sustainable management strategies. Our findings have shown that across both clones, ionic silver was more readily taken up than nanoparticulate silver and constituted the main source of stress and toxicity. Crucially, the effects of both treatments were found to be dose-dependent in the female clone, whereas the male clone exhibited a treatment-specific response. Since a genetic map for this plant species is available, the variability expressed by male and female clones of *P*. *nigra* could be further exploited with a genetic approach to identify key genes or mechanisms involved in the adaptive response.

Moreover, the in vitro tissue culture approach is not intended to be representative of the whole plant under field and greenhouse conditions, but rather serves as a preliminary investigation to explore plant responses to stress factors. Therefore, further comprehensive field experiments are necessary to validate these responses in natural systems.

## Figures and Tables

**Table 1:** Effects of AgNPs-cit-GSH (2.5 and 5 mg/L) and AgNO_3_ (2.5 and 5 mg/L), on Fresh weight, Dry weight, and Water content in poplar calli (clones 58-861 and Poli). Data represent the mean ± SD (*n* = 3). Different letters indicate statistically significant differences (*p* < 0.05, Duncan’s test). The analysis of variance for the effect of poplar clone, AgNPs-cit-GSH, and AgNO_3_ treatments, and their interaction is reported (* *p* < 0.05; ** *p* < 0.01; *** *p* < 0.001; ns not significant).

Clone	Treatment (mg/L)	Fresh Weight (g)	Dry Weight (g)	Water Content (%)
	0	0.39 ± 0.084 ^b^	0.019 ± 0.004 ^cd^	95.04 ^b^
58-861	AgNPs-cit-GSH 2.5	0.45 ± 0.080 ^b^	0.024 ± 0.58 ^b^	94.7 ^b^
	AgNPs-cit-GSH 5	0.51 ± 0.12 ^b^	0.025 ± 0.006 ^b^	95.1 ^b^
	AgNO_3_ 2.5	0.28 ± 0.075 ^c^	0.017 ± 0.005 ^de^	94 ^b^
	AgNO_3_ 5	0.41 ± 0.12 ^b^	0.024 ± 0.007 ^b^	94.1 ^b^
	0	1.04 ± 0.18 ^a^	0.034 ± 0.006 ^a^	96.7 ^a^
Poli	AgNPs-cit-GSH 2.5	0.44 ± 0.15 ^b^	0.023 ± 0.008 ^bc^	94.8 ^b^
	AgNPs-cit-GSH 5	0.27 ± 0.05 ^c^	0.015 ± 0.003 ^de^	94.3 ^b^
	AgNO_3_ 2.5	0.3 ± 0.08 ^c^	0.015 ± 0.004 ^e^	95 ^b^
	AgNO_3_ 5	0.45 ± 0.1 ^b^	0.018 ± 0.004 ^de^	95.9 ^b^
		Clone: ns	Clone: *	Clone: *
		TRT: ***	TRT: ***	TRT: **
		Clone × TRT: ***	Clone × TRT: ***	Clone × TRT: *

**Table 2:** Ag content in poplar calli (clones 58-861 and Poli) exposed for three weeks to AgNPs-cit-GSH (2.5 and 5 mg/L) and AgNO_3_ (2.5 and 5 mg/L). Data represent the mean ± SD (*n* = 3). Different letters indicate statistically significant differences (*p* < 0.05, Duncan’s test). The analysis of variance for the effect of poplar clone, AgNPs-cit-GSH and AgNO_3_ treatments, and their interaction is reported (*** *p* < 0.001).

Clone	Treatment (mg/L)	Ag Content (µg/g DW)
	0	<LOD
58-861	AgNPs-cit-GSH 2.5	7.79 ± 2.5 ^e^
	AgNPs-cit-GSH 5	5.05 ± 1.16 ^f^
	AgNO_3_ 2.5	375.8 ± 203.9 ^b^
	AgNO_3_ 5	490.4 ± 31.4 ^b^
	0	<LOD
Poli	AgNPs-cit-GSH 2.5	12.72 ± 2.65 ^d^
	AgNPs-cit-GSH 5	21.01 ± 4.2 ^c^
	AgNO_3_ 2.5	401.96 ± 49.35 ^b^
	AgNO_3_ 5	774.41 ± 1.14 ^a^
		Clone: ***
		TRT: ***
		Clone × TRT: ***

**Table 3:** The analysis of variance for the effect of poplar clone, AgNPs-cit-GSH and AgNO_3_ treatments, and their interaction on nutrient uptake (* *p* < 0.05; ** *p* < 0.01; *** *p* < 0.001).

Nutrient	Clone	Treatment (TRT)	Clone × TRT
Ca	***	***	***
Cu	***	***	***
K	***	***	***
Mg	***	***	***
Mn	***	***	**
Na	*	***	***
S	***	*	***
Zn	***	***	***

## Data Availability

The original contributions presented in this study are included in the article/Supplementary Material. Further inquiries can be directed to the corresponding author.

## Supplementary Materials

The following supporting information can be downloaded at https://www.mdpi.com/article/10.3390/plants14233560/s1, Table S1: Lipid peroxidation content (MDA), protein content and H^+^-ATPase activity; Table S2: Catalase (CAT), ascorbate peroxidase (APX), glutathione-S-transferase (GST); Table S3: macro and micronutrient content.
