# Supplementary material for: Sex-Related Differences in Physiological and Biochemical Responses of Populus nigra to Bifunctionalized Silver Nanoparticles and Silver Ions Exposure In Vitro
Source: Plants (Basel). 2025 Nov 21;14(23):3560. doi: 10.3390/plants14233560 (PMC12693857; doi:10.3390/plants14233560)
Supplement: Supplementary file 1 [file plants-14-03560-s001.zip › plants-3988792-supplementary.pdf]

Table S1. Lipid peroxidation content (MDA), protein content and H^+-ATPase activity in callus cultures of *Populus nigra* L. clones 58-861 and Poli exposed for 3 weeks to AgNPs-cit-GSH (2.5 and 5 mg/L) and $AgNO_3$ (2.5 and 5 mg/L) (± SD, n = 3). Different letters indicate significant differences (p ≤ 0.05, Duncan's test).

Clone	Treatment (mg/L)	MDA (nmol/g DW)	Protein content (mg/g DW)	ATPase activity (µmol Pi/min x mg prot)
	0	233.8±13.09[bc]	17.77±0.67[a]	0.0345±0.003[a]
**58-861**	AgNPs-cit-GSH 2.5	164.99±1.56[e]	15.36±0.58[b]	0.0295±0.004[bc]
	AgNPs-cit-GSH 5	206.32±9.3[d]	15.69±0.6[b]	0.0225±0.004[de]
	$AgNO_3$ 2.5	242.09±23.9[bc]	13.96±0.6[c]	0.0335±0.001[a]
	$AgNO_3$ 5	133.22±9.3[f]	14.62±0.34[bc]	0.0245±0.005[d]
	0	251.23±4.05[b]	8.13±1.05[e]	0.0265±0.002[cd]
**Poli**	AgNPs-cit-GSH 2.5	204.88±13.62[d]	13.8±1.96[c]	0.0325±0.002[ab]
	AgNPs-cit-GSH 5	221.06±31.35[cd]	10.57±1.28[d]	0.0198±0.002[e]
	$AgNO_3$ 2.5	251.29±18.16[b]	15.32±0.51[b]	0.0265±0.004[cd]
	$AgNO_3$ 5	301.78±9.6[a]	18.66±0.14[a]	0.026±0.003[cd]

Table S2. Catalase (CAT), ascorbate peroxidase (APX), glutathione-S-transferase (GST) in callus cultures of *Populus nigra* L. clones 58-861 and Poli exposed for 3 weeks to AgNPs-cit-GSH (2.5 and 5 mg/L) and $AgNO_3$ (2.5 and 5 mg/L) (± SD, n = 3). Different letters indicate significant differences (p ≤ 0.05, Duncan's test).

Clone	Treatment (mg/L)	APX (µmol AsA/mg prot x min)	CAT (µmol H_2O_2/mg prot x min)	GST (mM CDNB/mg prot x min)
	0	13.8±2.73[c]	0.132±0.002[ef]	0.219±0.034[d]
**58-861**	AgNPs-cit-GSH 2.5	11.04±0.02[d]	0.107±0.01[f]	0.236±0.004[d]
	AgNPs-cit-GSH 5	11.12±0.46[d]	0.155±0.007[de]	0.350±0.003[bc]
	$AgNO_3$ 2.5	5.98±0.26[f]	0.148±0.03[e]	0.331±0.033[c]
	$AgNO_3$ 5	9.57±0.09[e]	0.163±0.008[de]	0.358±0.045[bc]
	0	20.17±0.71[a]	0.33±0.009[a]	0.358±0.000[bc]
**Poli**	AgNPs-cit-GSH 2.5	11.46±0.68[d]	0.164±0.03[de]	0.397±0.051[b]
	AgNPs-cit-GSH 5	16.31±0.12[b]	0.191±0.04[cd]	0.471±0.053[a]
	$AgNO_3$ 2.5	11.55±0.63[d]	0.211±0.02[c]	0.386±0.025[bc]
	$AgNO_3$ 5	10.23±1.12[de]	0.253±0.02[b]	0.328±0.009[c]

Table S3. Calcium (Ca), Copper (Cu), Potassium (K), Magnesium (Mg), Manganese (Mn), Sodium (Na), Sulfur (S), and Zinc (Zn) content in callus cultures of *Populus nigra* L. clones 58-861 and Poli exposed for 3 weeks to AgNPs-cit-GSH (2.5 and 5 mg/L) and $AgNO_3$ (2.5 and 5 mg/L) (± SD, n = 3). Different letters indicate significant differences ($p \leq 0.05$, Duncan's test).

**Clone**	**Treatment (mg/L)**	**Ca (mg/g DW)**	**Cu (µg/g DW)**	**K (mg/g DW)**	**Mg (mg/g DW)**	**Mn (mg/g DW)**	**Na (mg/g DW)**	**S (mg/g DW)**	**Zn (µg/g DW)**
**58-861**	0	25.23 ± 0.57^{c}	12.49 ± 0.9^{c}	297.96 ± 19^{b}	12.44 ± 1.12^{b}	1.36 ± 0.06^{ab}	17.04 ± 1.17^{a}	31.88 ± 0.54^{de}	825.35 ± 38.04^{c}
	AgNPs-cit-GSH 2.5	24.72 ± 1.28^{c}	11.33 ± 1.33^{c}	181.32 ± 9.7^{d}	9.16 ± 0.11^{bc}	1.11 ± 0.02^{bc}	10.16 ± 1.76^{cd}	31.17 ± 2.41^{de}	759.51 ± 41.45^{d}
	AgNPs-cit-GSH 5	15.47 ± 0.31^{d}	6.62 ± 0.33^{d}	97.82 ± 1.73^{f}	4.20 ± 0.12^{e}	0.71 ± 0.02^{e}	6.36 ± 0.71^{e}	24.16 ± 0.85^{f}	446.6 ± 1.85^{g}
	$AgNO_3$ 2.5	27.09 ± 2.33^{bc}	10.76 ± 0.32^{c}	128.08 ± 10.83^{ef}	6.14 ± 0.69^{cde}	1.27 ± 0.09^{abc}	9.92 ± 0.43^{cd}	35.01 ± 7.63^{cd}	660.74 ± 56.09^{e}
	$AgNO_3$ 5	19.01 ± 1.92^{d}	13.08 ± 0.72^{c}	98.19 ± 5.73^{f}	4.68 ± 0.36^{de}	0.86 ± 0.06^{de}	7.56 ± 0.07^{de}	28.11 ± 1.05^{ef}	436.16 ± 7.5^{g}
**Poli**	0	30.06 ± 5.18^{b}	12.7 ± 2.6^{c}	386.44 ± 30.1^{a}	28.55 ± 6.36^{a}	1.42 ± 0.23^{a}	13.5 ± 2.66^{b}	37.44 ± 0.16^{bc}	1393.5 ± 38.6^{a}
	AgNPs-cit-GSH 2.5	25.21 ± 2.95^{c}	16.52 ± 1.17^{b}	259.46 ± 19.67^{c}	7.93 ± 0.43^{cde}	1.06 ± 0.12^{cd}	7.77 ± 0.15^{de}	34.71 ± 0.35^{cd}	815.8 ± 57.5^{cd}
	AgNPs-cit-GSH 5	26.98 ± 0.89^{bc}	13.63 ± 2.12^{bc}	227.24 ± 36.41^{c}	8.10 ± 1.23^{cde}	1.19 ± 0.12^{abc}	10.68 ± 1.58^{bc}	41.92 ± 2.98^{ab}	919.9 ± 18.5^{b}
	$AgNO_3$ 2.5	25.24 ± 1.62^{c}	25.41 ± 0.84^{a}	159.27 ± 19.08^{de}	8.18 ± 0.93^{cde}	1.36 ± 0.02^{ab}	11.12 ± 2.03^{bc}	40.36 ± 1.71^{ab}	593.7 ± 39.03^{f}
	$AgNO_3$ 5	38.44 ± 1.31^{a}	22.61 ± 4.23^{a}	158.4 ± 12.15^{de}	8.49 ± 0.78^{cd}	1.33 ± 0.27^{ab}	11 ± 2.23^{bc}	42.78 ± 1.12^{a}	843.3 ± 17.9^{c}
